# SCHIZORIZA domain–function analysis identifies requirements for its specific role in cell fate segregation

**DOI:** 10.1093/plphys/kiad456

**Published:** 2023-08-16

**Authors:** Renan Pardal, Ben Scheres, Renze Heidstra

**Affiliations:** Cluster of Plant Developmental Biology, Laboratory of Molecular Biology, Wageningen University & Research, 6708 PB, Wageningen, The Netherlands; Cluster of Plant Developmental Biology, Laboratory of Molecular Biology, Wageningen University & Research, 6708 PB, Wageningen, The Netherlands; Cluster of Plant Developmental Biology, Laboratory of Molecular Biology, Wageningen University & Research, 6708 PB, Wageningen, The Netherlands

## Abstract

Plant development continues postembryonically with a lifelong ability to form new tissues and organs. Asymmetric cell division, coupled with fate segregation, is essential to create cellular diversity during tissue and organ formation. Arabidopsis (*Arabidopsis thaliana*) plants harboring mutations in the *SCHIZORIZA* (*SCZ*) gene display fate segregation defects in their roots, resulting in the presence of an additional layer of endodermis, production of root hairs from subepidermal tissue, and misexpression of several tissue identity markers. Some of these defects are observed in tissues where *SCZ* is not expressed, indicating that part of the SCZ function is nonautonomous. As a class B HEAT-SHOCK TRANSCRIPTION FACTOR (HSFB), the SCZ protein contains several conserved domains and motifs. However, which domain(s) discriminates SCZ from its family members to obtain a role in development remains unknown. Here, we investigate how each domain contributes to SCZ function in Arabidopsis root patterning by generating altered versions of SCZ by domain swapping and mutation. We show that the SCZ DNA-binding domain is the main factor for its developmental function, and that SCZ likely acts as a nonmotile transcriptional repressor. Our results demonstrate how members of the HSF family can evolve toward functions beyond stress response.

## Teaser

Domain–function analysis shows that evolution of the heat-shock transcription factor family gene *SCHIZORIZA* toward a function in development resides predominantly in its DNA-binding domain.

## Introduction

Different from animals, plants display indeterminate growth and development, producing new organs during their whole life cycle. This continuous development employs asymmetric cell division as a fundamental mechanism to generate diversity and patterning (Knoblich 2008; Pillitteri et al. 2016). It is allowed by the existence of 2 populations of stem cells at the shoot and root extremities, which lie in the center of the shoot apical meristem and root apical meristem. The mitotically active stem cells surround the scarcely dividing organizer cells required for their maintenance, forming the stem cell niche (SCN) (van den Berg et al. 1997; Schoof et al. 2000; Baurle and Laux 2003). In the Arabidopsis (*Arabidopsis thaliana*) root meristem, stereotyped asymmetric cell divisions of the stem cells, also called initials, originate the radial organization of the Arabidopsis root with its defined cell files and tissue layers. Initials distal to the quiescent center (QC) divide to form the columella root cap consisting of ordered columns of cells accumulating starch-laden amyloplasts (Dolan et al. 1993).

Arabidopsis plants mutant for the *SCHIZORIZA* (*SCZ*, *AT1G46264*) gene display several defects in their developing roots. Morphologically, these mutant roots are characterized by the absence of a QC, a disorganized columella root cap, the subepidermal emergence of root hairs, and an extra endodermal layer. This additional layer is a consequence of an aberrant periclinal division undergone by the ground tissue initial cells during embryogenesis, which originates 2 initial daughters. The inner daughter cell undergoes a second round of periclinal division, forming the additional endodermal tissue layer characterized by expression of the SCARECROW (SCR) endodermis marker (ten Hove et al. 2010). Besides the aberrant morphology, additional tissue identity markers are misexpressed. The cortical markers, *Co2* and *Co3*, are not expressed, indicating loss of cortical identity of the outer ground tissue layer. The mutant cortex now produces root hairs and expresses the epidermal *GL2* promoter activity, suggesting the acquirement of epidermal identity characteristics. Furthermore, the lateral root cap–expressed *SMB* gene becomes expressed in the *scz* mutant epidermis (ten Hove et al. 2010). Together, these observations implicate SCZ in the segregation of different root tissue fates. In situ hybridization experiments demonstrated that the *SCZ* mRNA localizes to the QC, ground tissue, and stele. Given that *scz* mutants also display defects in regions outside of the *SCZ* expression domain, part of the SCZ function must be exerted in a noncell-autonomous way (Mylona et al. 2002; Pernas et al. 2010; ten Hove et al. 2010).

SCZ belongs to the family of HEAT-SHOCK TRANSCRIPTION FACTORS (HSFs) and is also known as HSFB4. HSFs have an essential role in the ability of plants to respond to abiotic stresses, such as drought, salinity, and heat stress, that affect their growth and development. As a result, the expression of stress-responsive genes, including heat-shock protein (HSP)–type chaperones, is induced to counteract negative effects on protein folding, assembly, translocation, and degradation (Guo et al. 2016). Unlike typical HSF genes like *HSFA1s*, *HSFA2*, *HSFB1*, and *HSFB2b*, expression of *SCZ* is barely affected by heat treatment (von Koskull-Döring et al. 2007; Scharf et al. 2012) and appears to be adopted for developmental control (Mylona et al. 2002; Pernas et al. 2010; ten Hove et al. 2010; Begum et al. 2013; Olmo et al. 2020). However, what discriminates SCZ and its role in development from the other HSFB class members remains unknown.

**Figure 1.**
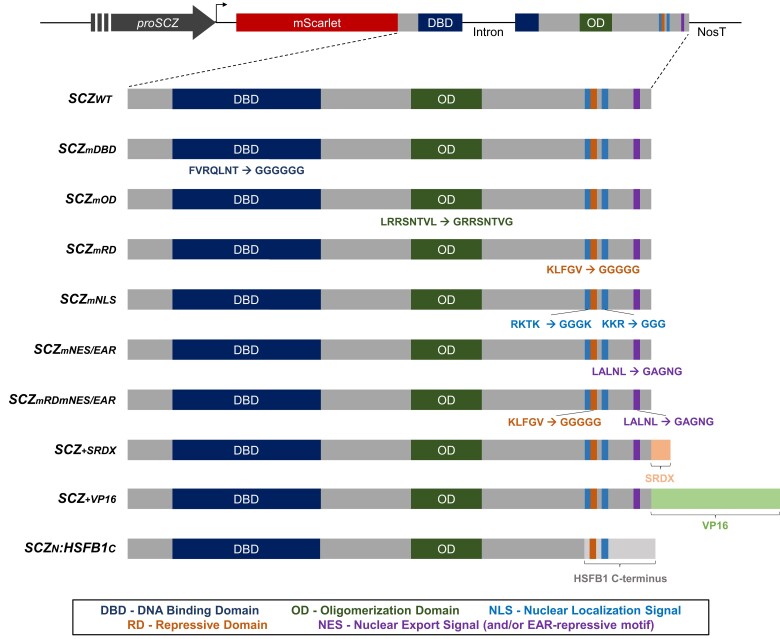
Schematic representation of SCZ variants used in this study. On top, a general outline of the transcriptional cassette used to transform *scz-2* plants. The WT cassette consists of a ∼4.2 kb *SCZ* promoter (*proSCZ*), upstream of *mScarlet* fused to the genomic sequence of *SCZ*, which is followed by the *Nopaline Synthase Terminator* sequence (*NosT*). Except for the promoter, all components of the construct are proportionally represented. Below, a scheme of each SCZ protein variant, specifying either the amino acid replacements or the swap/addition of motifs. The changes in amino acid sequences are colored according to the location and domain of the mutation, with the original sequence followed by the mutated amino acid stretch. Added or replaced domains are indicated by a bracket above the domain name.

The HSF group of transcription factors contains several conserved domains and motifs in their sequence (Fig. 1; Supplemental Figs. S1 and S2). The most highly conserved domain of HSFs between different organisms is the N-terminal DNA-binding domain (DBD), which contains a central helix–turn–helix motif necessary for the recognition and binding of the heat-shock element sequences in the DNA of target genes (HSEs) (Vuister et al. 1994; Schultheiss et al. 1996; Littlefield and Nelson 1999; Nover et al. 2001; Scharf et al. 2012; Guo et al. 2016). Downstream of the DBD, connected by a flexible linker of variable size, is located the oligomerization domain (OD). This domain consists of 2 parts (A and B), harbors a heptad of hydrophobic amino acids, and is essential for the formation of homotrimers and heterooligomers between HSFs (Sorger and Nelson 1989; Hubel et al. 1995; Orosz et al. 1996; Scharf et al. 1998; Chan-Schaminet et al. 2009). The structure of the OD is the basis for the classification of HSFs in plants: while class A HSFs have a 21-amino acid stretch between OD parts A and B, the class C HSFs have only 7 amino acids separating them. On the other hand, class B HSFs—to which SCZ belongs—do not present a linker between OD-A and OD-B, therefore displaying a shorter OD. The HSFs also contain a nuclear localization signal (NLS) that may be monopartite or bipartite, and many HSFs also display a nuclear export signal (NES) that is generally located at the C-terminal region (Nover et al. 2001; Scharf et al. 2012; Guo et al. 2016). This C-terminal region of HSFs is quite variable: whereas most HSF-As present one or more AHA transcription activation domains at this region, most HSF-Bs display a tetrapeptide LFGV shown to act as a repressive domain (RD) instead (Nover et al. 2001; Ikeda and Ohme-Takagi 2009; Zhu et al. 2012).

In this study, we test the attributed roles of each SCZ domain and relate these to the phenotypes observed in *scz* mutants. This way, we investigate whether SCZ is indeed a transcriptional repressor, and whether protein mobility is required for its nonautonomous function in fate segregation. Furthermore, we show that 2 stress-related *HSFBs* can partially replace SCZ. Thus, we identify the main domain responsible for functionally differentiating SCZ, with respect to its role in root patterning, from stress-related class B HSFs.

## Results

SCZ, a B-class heat-shock transcription factor also known as HSFB4, is 1 among the 21 transcription factors belonging to the Arabidopsis HSF family that appears to be adopted for a role in root development. Similar to all HSFs, the SCZ protein contains an N-terminal DBD followed by an OD sequence. In addition, SCZ displays in its C-terminal domain a bipartite NLS that partially overlaps with the LFGV-designated RD. Located downstream to the NLS/RD is a presumed NES that overlaps with a putative EAR motif (consensus sequence LxLxL), which we, therefore, label as NES/EAR (Fig. 1) (Ohta et al. 2001; Ikeda and Ohme-Takagi 2009; ten Hove et al. 2010; Ikeda et al. 2011; Scharf et al. 2012; Zhu et al. 2012; Guo et al. 2016). Importantly, these domains and motifs were mapped in the SCZ protein based solely on homology, without experimental data supporting their function.

**Figure 2.**
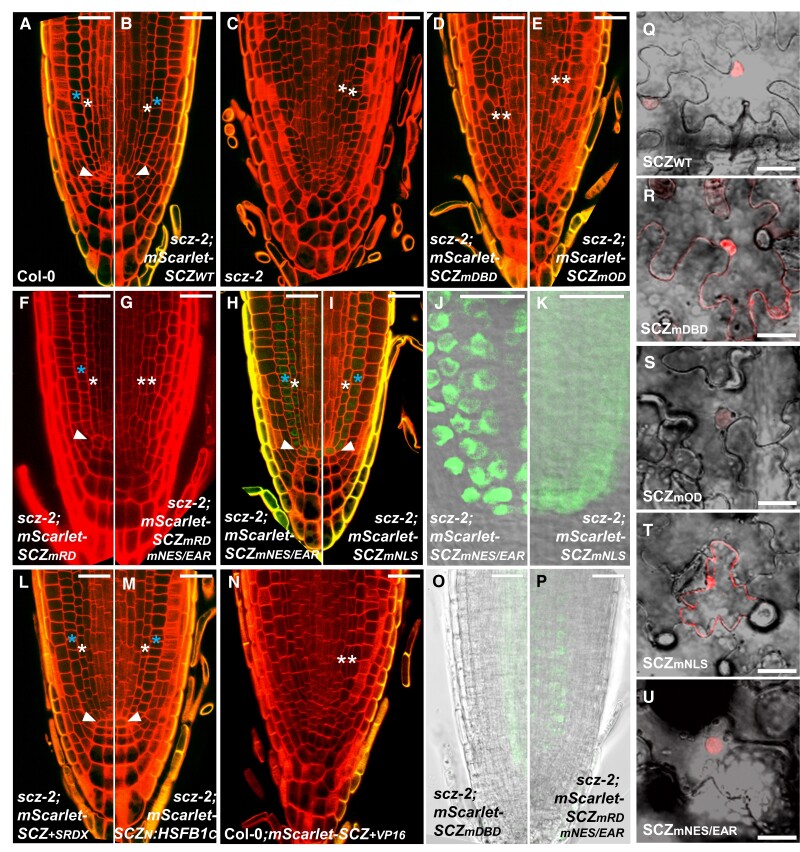
Complementation of *scz-2* root defects by SCZ domain variants. **A)** WT (Col-0). **B)***scz-2* complemented with *proSCZ::mScarlet-SCZ_WT_*. **C)***scz-2*. **A to M)** Root tips of *scz-2* plants expressing *proSCZ::mScarlet-SCZ_mDBD_***D)**, *proSCZ::mScarlet-SCZ_mOD_***E)**, *proSCZ::mScarlet-SCZ_mRD_***F)**, *proSCZ::mScarlet-SCZ_mRDmNES/EAR_***G)**, *proSCZ::mScarlet-SCZ_mNES/EAR_***H**, **J)**, *proSCZ::mScarlet-SCZ_mNLS_***I**, **K)**, *proSCZ::mScarlet-SCZ _+ SRDX_***L)**, and *proSCZ::mScarlet-SCZ_N_:HSFB1_C_***M)**. **J)** and **K)** represent siblings of plants imaged in **H)** and **I)**, respectively. **N)** Root tip of Col-0 expressing *proSCZ::mScarlet-SCZ_+VP16_*. **O)***scz-2;proSCZ::mScarlet-SCZ_mDBD_* root displaying weak and diffuse mScarlet fluorescence. **P)***scz-2;proSCZ::mScarlet-SCZ_mRDmNES/EAR_* roots displaying nuclear mScarlet fluorescence. 5dag root tips were subject to the ClearSee protocol and stained with Direct Yellow 96 **A to E**, **H**, **I**, **L to N)**; freshly stained with PI **F**, **G)**; or nonstained **J**, **K**, **O**, **P)**. mScarlet fluorescence, when visible, is displayed in false green **A to P)**, and cell walls are displayed in false red **A to I**, **L to N)**. White arrowhead points to the WT-like QC. White asterisk marks the single (WT) or double (mutant) endodermis. Blue asterisk marks the cortex in WT and complemented roots. **Q to U)** Subcellular localization in cells of transiently transformed *N. benthamiana* leaves of mScarlet-tagged SCZ_WT_**Q)**, SCZ_mDBD_**R)**, SCZ_mOD_**S)**, SCZ_mNLS_**T)**, and SCZ_mNES/EAR_**U)** accumulation. *SCZ* variants were expressed under control of the *35S* promoter **Q to U)**. Scale bar for all panels = 25 *μ*m.

The *scz-2* mutant contains a 1-base pair deletion at the C-terminus of *SCZ*, leading to a frameshift and absence of the final 42 amino acids in the encoded protein. This truncated protein lacks the RD, the second portion of the NLS and the NES/EAR motif (Supplemental Fig. S1). Given the identical phenotypes observed for mutations in more 5′ regions of the gene, the *scz-2* mutation appears sufficient to abolish SCZ function (ten Hove et al. 2010), thereby justifying the use of this mutant for our studies. To understand to what extent each conserved domain/motif contributes to SCZ function in root patterning, SCZ variants containing amino acid replacements were generated. Both in the larger domains like the DBD and OD, as in the shorter RD, NLS, and NES/EAR motifs, key amino acids—highly conserved across species or even kingdoms—were replaced by glycine residues generating the mDBD, mOD, mRD, mNLS, and mNES/EAR variants, respectively (Fig. 1; Supplemental Figs. S1 and S2). In addition, we tagged each variant with a fluorescent protein translationally fused to its N-terminal extremity. Previous translational fusions of GFP to SCZ functionally complemented the *scz-2* mutant but failed to produce a consistent fluorescence signal in Arabidopsis (ten Hove et al. 2010). mScarlet was chosen to replace GFP because it was the brighter fluorescent protein available at the time these experiments were initiated (Bindels et al. 2017). Expression of the tagged variants was under the control of a 4.2-kilobase (kb) *SCZ* endogenous promoter sequence (*proSCZ*) (Fig. 1). The *mScarlet*-tagged variants were transformed into *scz-2* plants, and their progeny was analyzed in detail by confocal microscopy for protein localization and phenotypic rescue (Fig. 2).

### DBD and OD are essential for SCZ function

As a control, *scz-2* plants were transformed with the construct harboring the *mScarlet*-tagged wild-type *SCZ* version (*mScarlet-SCZWT*, Fig. 1), and transgenic lines were selected. We observed a morphologically distinguishable QC, a single endodermal layer, and an absence of subepidermal root hairs, indicating full complementation of the *scz-2* mutant phenotype (Fig. 2, A and B; Supplemental Fig. S3), despite the absence of fluorescent signal.

Surprisingly, variants containing mutations in the RD, NES/EAR, or NLS were all able to phenotypically rescue *scz-2* (Fig. 2, B, F, H, and I). Similar to the mScarlet-SCZWT, the mScarlet-SCZ_mRD_ rescued *scz-2* defects without showing fluorescent signal (Fig. 2F). In *scz-2;mScarlet-SCZ_mNES/EAR_* and *scz-2;mScarlet-SCZ_mNLS_* transgenics, mScarlet fluorescence could be detected, albeit weak (Fig. 2, H to K). The only SCZ variants incapable of rescuing the mutant root pattern were *mScarlet-SCZ_mDBD_* and *mScarlet-SCZ_mOD_*, which harbor mutations in the DBD and OD, respectively. The roots of these plants displayed a morphology undistinguishable from the *scz-2* mutant, exhibiting a disorganized columella, absence of QC, double endodermis, and subepidermal root hairs (Fig. 2, C to E).

Weak and diffuse fluorescent signal throughout the cells was observed in mScarlet-SCZ_mDBD_ roots (Fig. 2, D and O), whereas no signal was observed in any mScarlet-SCZ_mOD_ lines (Fig. 2E), making it unclear whether the failed complementation was due to a lack of protein production or due to the mutation per se. Even though complementation by mScarlet-SCZWT showed that detection of mScarlet fluorescence is not a prerequisite for phenotypic rescue (Fig. 2B), we decided to investigate protein localization and accumulation by ectopic expression in *Nicotiana benthamiana.* To this end, constructs were generated in which the same *mScarlet*-tagged *SCZWT*, *SCZ_mDBD_*, and *SCZ_mOD_* variants were now placed under the control of the strong and constitutive cauliflower mosaic virus (CaMV) *35S* promoter. These constructs were then transiently expressed in *N. benthamiana* leaves by agroinfiltration, and protein accumulation was examined by confocal scanning laser microscopy. All three mScarlet-tagged SCZWT, SCZ_mDBD_, and SCZ_mOD_ versions accumulated in the nucleus; however, mScarlet-SCZ_mDBD_ also accumulated in the cytosol (Fig. 2, Q to S). These results support the idea that the lack of phenotypic rescue observed in *scz-2;mScarlet-SCZ_mDBD_* and *scz-2;mScarlet-SCZ_mOD_* lines was indeed caused by the disruption of DBD or OD function and not due to lack of protein production.

Together, these results on single-domain mutation variants indicate that the DBD and OD domains are essential for SCZ function in root patterning. However, a combinatorial role of RD, NLS, and NES/EAR domains for SCZ function cannot be discarded at this point.

### SCZ nuclear localization is not fully NLS dependent

Both the *mScarlet*-tagged SCZ_mNES/EAR_ and the SCZ_mNLS_ variants rescued the *scz-2* mutant defects. mScarlet-SCZ_mNES/EAR_ displayed a strictly nuclear accumulation, suggesting that nuclear expression is required for SCZ function, and that this putative NES/EAR is not an actual export signal (Fig. 2, H and J). mScarlet-SCZ_mNLS_ accumulation appears diffuse throughout the cells in the *SCZ* expression domain (Fig. 2I), and higher magnification confocal images show that SCZ_mNLS_ is not excluded from the nucleus (Fig. 2K). This indicates that the mutation of the NLS is insufficient to abolish the SCZ nuclear import. Since none of our complementation lines expressing mScarlet-SCZWT produced detectable fluorescence (Fig. 2B), it was unclear what the original subcellular localization of SCZ was. Consequently, it was not possible to discern if the mutations in the NLS or NES/EAR actually disrupted SCZ localization.

To clarify SCZ protein variant localization, constructs were generated for transient expression in *N. benthamiana*. The same *mScarlet*-tagged *SCZ_mNES/EAR_* and *SCZ_mNLS_* were placed under the control of the *35S* promoter, and after transformation into *N. benthamiana* leaves, their protein accumulation pattern was compared to mScarlet-SCZWT. Whereas mScarlet-SCZWT displays an exclusively nuclear accumulation, mScarlet-SCZ_mNLS_ localizes to the cytosol and nucleus (Fig. 2, Q and T). Thus, even though a mutated NLS increased cytosolic presence, it did not completely prevent mScarlet-SCZ_mNLS_ from going into the nucleus, as was also observed in Arabidopsis roots. This indicates that the C-terminal bipartite NLS is not the sole motif responsible for SCZ nuclear localization. Accordingly, mScarlet-SCZ_mDBD_ also displayed increased cytosolic accumulation compared to mScarlet-SCZ_WT_ (Fig. 2, Q and R), suggesting that the DBD also contributes to SCZ nuclear localization. The presence of the DBD domain may explain why mScarlet-SCZ_mNLS_ was not excluded from the nucleus and therefore able to rescue *scz-2* root defects. Furthermore, mScarlet-SCZ_mOD_ accumulated exclusively in the nucleus (Fig. 2E), indicating that the OD does not play a role in SCZ subcellular localization.

Comparing mScarlet-SCZ_mNES/EAR_ to mScarlet-SCZWT shows that both display a strict nuclear accumulation (Fig. 2, Q and U). It is, therefore, not possible to state that the NES/EAR motif indeed promotes SCZ nuclear export. Considering that the annotated NES overlaps with the EAR motif consensus LxLxL (Ohta et al. 2001; ten Hove et al. 2010), this region may therefore confer transcriptional repressive activity instead.

Together, the above results indicate that SCZ localizes primarily to the nucleus, and that an exclusively nuclear localization is sufficient for its function in root patterning. In addition, the results suggest that both the NLS and DBD play a role in promoting SCZ nuclear localization. Furthermore, both mScarlet-SCZ_mNES/EAR_ and mScarlet-SCZ_mNLS_ accumulation in roots colocalize with the previously reported *SCZ* mRNA expression domain: QC, ground tissue, and stele (ten Hove et al. 2010). This strongly suggests that SCZ does not move to the outer layers to exert its function in the fate segregation of epidermis, lateral root cap, and columella.

### SCZ acts as a transcriptional repressor in vivo

As an HSFB, SCZ is expected to act as a transcriptional repressor. Two other members of the SCZ clade were previously shown to act as transcriptional repressors in an RD-dependent manner (Ikeda and Ohme-Takagi 2009; Ikeda et al. 2011; Scharf et al. 2012; Zhu et al. 2012; Guo et al. 2016). However, both HSFB3 and SCZ failed to display repressive activity in 2 independent luciferase assays (Ikeda et al. 2011; Zhu et al. 2012). In addition, here we observed that upon mutation of the RD, the SCZ protein remained functional in the way that mScarlet-SCZ_mRD_ was able to rescue *scz-2* root defects (Fig. 2F). This suggests that either SCZ is not a repressor or that SCZ repressive activity is not (only) dependent on the RD motif. To provide evidence that SCZ does act as a repressor, 3 additional mScarlet-tagged SCZ fusions were generated. In the first version, a strong repressive EAR (ERF-associated amphiphilic repression) motif, called SRDX (Ohta et al. 2001; Hiratsu et al. 2002; Hiratsu et al. 2003), was added to the C-terminus of SCZ_WT_ creating SCZ + SRDX (Fig. 1). The SRDX motif, consisting of only 12 amino acids, has been described in the literature to convert transcriptional activators into repressors (Hiratsu et al. 2003). In the second version, the C-terminal region of SCZ was replaced by the C-terminal region of HSFB1 from Arabidopsis, creating SCZN:HSFB1C (Fig. 1). HSFB1 was previously shown to act as a repressor (Ikeda et al. 2011; Zhu et al. 2012). The SCZN:HSFB1C version contains the HSFB1-derived NLS and RD, but not a NES/EAR domain, since HSFB1 lacks one. Different from what is observed in SCZ, the NLS from HSFB1 is monopartite (Supplemental Figs. S1 and S2D). The RDs from HSFB1 and SCZ have an identical amino acid sequence (Supplemental Fig. S2C). The third variant was the fusion of the strong transcriptional activator VP16 to the C-terminus of SCZWT creating SCZ + VP16 (Fig. 1). The VP16 domain was reported to efficiently convert transcriptional repressors into activators, even resulting in plants with knockout-like phenotype (Fujiwara et al. 2014). The coding region of these variants was fused to *mScarlet* at the N-terminus and expressed from the same 4.2-kb *SCZ* endogenous promoter (*proSCZ*) as described above. The *mScarlet*-*SCZ + SRDX* and *mScarlet*-*SCZN:HSFB1C* fusions were tested for complementation of the *scz-2* mutant phenotype, whereas the *mScarlet*-*SCZ + VP16* variant was transformed into Col-0 WT plants (Fig. 2, L to N).

The mScarlet-SCZ + SRDX and mScarlet-SCZN:HSFB1C variants completely rescued *scz-2* root defects (Fig. 2, L and M). Apparently, either the replacement of the C-terminus of SCZ for the C-terminal repressor region of HSFB1 or the addition of the strong repressive SRDX motif did not alter the functions ascribed to the SCZ protein. On the other hand, in 6 out of 13 transgenic lines, WT plants expressing mScarlet-SCZ + VP16 displayed all root defects present in *scz-2* plants: double endodermis, disorganized columella cells, lack of QC (Fig. 2N), and root hair defects (Supplemental Fig. S4), indicating alleviation of the repressive activity of this protein variant. These results are consistent with the notion that SCZ acts as a repressor.

In the SCZ protein, besides the RD, there is a putative EAR motif overlapping with the mapped NES, making it conceivable that 1 repressive motif might compensate for the loss of the other, hence explaining that mScarlet-SCZ_mRD_ was able to rescue *scz-2* root defects (Fig. 2F). To test this hypothesis, an mScarlet-tagged variant was generated, in which both the RD and NES/EAR were mutated, creating mScarlet-SCZ_mRDmNES/EAR_ (Fig. 1). If our hypothesis is correct, this variant should not be functional and, therefore, unable to rescue *scz-2* root defects. Phenotypic analysis of transgenic *scz-2* expressing mScarlet-SCZ_mRDmNES/EAR_ showed that this variant was indeed unable to rescue *scz-2* mutant root defects at any level (Fig. 2G), despite the nuclear presence of protein, as evidenced by the mScarlet fluorescence (Fig. 2P). Altogether, these results support the role of SCZ as a transcriptional repressor. In addition, they suggest that the presence of either the RD or the NES/EAR motif is sufficient to confer a repressive activity to SCZ in vivo.

### The DBD is the key to SCZ functional specificity

**Figure 3.**
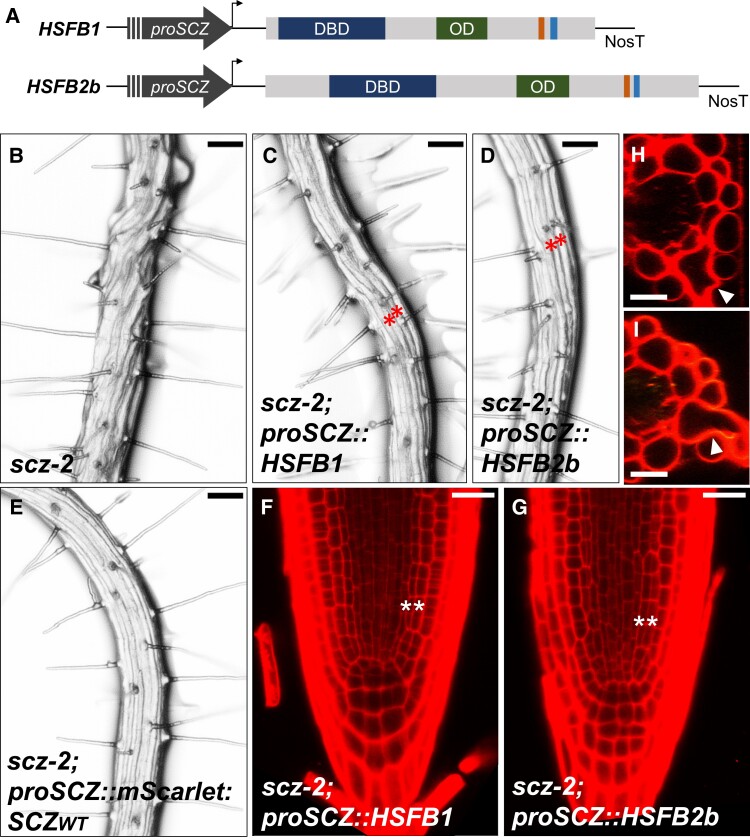
Partial complementation of *scz-2* by ectopic *HSFB1* and *HSFB2b* expression. **A)** Schematic representation of the constructs containing either *HSFB1*- or *HSFB2b*-coding sequences, under control of *proSCZ*. Indicated are the DBD in dark blue, OD in green, RD in orange, and NLS in light blue. **B to E)** Bright field images of the mature zone of the roots of *scz-2***B)**, *scz-2* expressing *proSCZ::HSFB1***C)**, *scz-2* expressing *proSCZ::HSFB2b***D)**, and *scz-2* expressing *proSCZ::mScarlet-SCZ_WT_***E)**. The latter exhibits the WT epidermal morphology and root hair pattern. **F**, **G)** Confocal images of the root tip of *scz-2* expressing *proSCZ::HSFB1***F)** or *proSCZ::HSFB2b***G)**. **H**, **I)** Digital cross-section of *scz-2;proSCZ::HSFB1***H)** and *scz-2;proSCZ::HSFB2b***I)** root showing a subepidermal root hair emerging between 2 epidermal cells (white arrowheads). For confocal imaging, fresh 5dag roots were stained with PI. Red asterisks mark adjacent root hair files. White asterisks mark double endodermis. Scale bar = 100 *μ*m **B to E)** or 25 *μ*m **F to I)**.

The complete rescue of the *scz-2* root defects by the mScarlet-tagged SCZN:HSFB1C variant (Fig. 2M) made us question whether the SCZ protein sequence holds specific properties that underlie its function in root patterning. Considering that the C-terminal region of both proteins greatly differs in sequence, apart from the RD consensus sequence (Supplemental Figs. S1 and S2C), it is remarkable that they are exchangeable. According to data retrieved from the Root Expression Atlas (eFP Browser), none of the *HSFBs* are expressed in the same domain as *SCZ* in Arabidopsis roots (Supplemental Fig. S5) (Brady et al. 2007). These observations led to the hypothesis that the specific role of SCZ in root patterning is due to its expression domain. In that case, expressing another class B HSF in the *SCZ* expression domain should rescue the *scz-2* root defects. To test this, the CDS of *HSFB1* and *HSFB2b* were cloned and placed under the control of *proSCZ* (Fig. 3A). When all HSFBs are aligned together, HSFB1 is the one sharing the highest percentage of identical amino acid residues with SCZ in relation to the alignment length (39.34%), whereas HSFB2b shares 37.22% of amino acid identity with SCZ (Supplemental Fig. S2E). In addition, these HSFBs have been shown to act as repressors in at least 2 independent experimental setups (Ikeda et al. 2011; Zhu et al. 2012). Each construct was transformed into *scz-2*, and transgenic progeny was tested for complementation of root defects. Comparing the morphological improvement of the root surface and the meristem organization, we selected the best complementing line for each genotype for detailed analysis. These analyses were conducted on segregating transgenic lines; therefore, a maximum of ∼75% of the roots displayed improved morphology for a particular line.

First, we imaged the mature zone of the root in *scz-2*, scz-2;*proSCZ::HSFB1*, and *scz-2;proSCZ::HSFB2b* lines. The epidermal cell files that give the twisted appearance in *scz-2* mutant roots (Fig. 3B) appear organized and easily traceable in scz-2;*proSCZ::HSFB1* and *scz-2;proSCZ::HSFB2b* roots. This phenotype segregated as a dominant trait, thereby suggesting at least some level of complementation (Fig. 3, C and D). However, these roots do exhibit adjacent files of hair cells (Fig. 3, C and D, red stars). Additionally, subepidermal root hairs were occasionally observed in both scz-2;*proSCZ::HSFB1* and *scz-2;proSCZ::HSFB2b* complementing lines (Fig. 3, H and I, white arrowheads). This is unlike *scz-2* plants complemented with mScarlet-tagged SCZWT, which display an epidermal pattern undistinguishable from WT, where files of hair cells are always separated by 1 or more files of nonhair cells (Fig. 3E; Supplemental Fig. S4A).

Next, we examined the meristematic zone, comparing columella organization, overall meristem anatomy, and endodermal file number. From all roots of the best complementing scz-2;*proSCZ::HSFB1* and *scz-2;proSCZ::HSFB2b* lines with an improved epidermal patterning, 80% and 75%, respectively, display a WT-like columella organization showing well-organized cell columns (Figs. 2A and 3, F to G; Supplemental Table S1). To score meristem anatomy, we aimed to distinguish between the meristem organization of the WT Arabidopsis, which represents a so-called closed meristem, where all tissue files originate from initial cells adjacent to the QC. This contrasts with the *scz* mutant root meristem that resembles an open meristem, where it appears that files of cells from the meristem continue into the root cap and, as such, presents an easily scorable phenotype (Supplemental Fig. S6) (Clowes 1981, 2000). We found that from all roots of the best complementing scz-2;*proSCZ::HSFB1* and *scz-2;proSCZ::HSFB2b* lines with an improved epidermal patterning, 100% and 75%, respectively, exhibit a closed meristem phenotype (Figs. 2A and 3, F to G; Supplemental Table S1). However, all of the scz-2;*proSCZ::HSFB1* and *scz-2;proSCZ::HSFB2b* roots still possess a double-layered endodermis, characteristic of the *scz* mutant phenotype (Fig. 3, F to G; Supplemental Table S1). Together, we observed that the root meristem morphology of scz-2;*proSCZ::HSFB1* and *scz-2;proSCZ::HSFB2b* lines was only partially rescued. These results refute the hypothesis that the SCZ function resides uniquely in its expression pattern. Instead, they highlight the relevance of the SCZ N-terminal protein sequence that must contain characteristics not shared by HSFB1 and HSFB2b, thereby allowing the SCZN:HSFB1C fusion to fully rescue *scz-2* root defects (Fig. 2M). In this sense, the most conserved domain across HSFBs is the DBD, displaying a percentage of identity as high as 80.65% (HSFB2a versus HSFB2b). SCZ shares 72.04% and 77.42% amino acid identity with HSFB1 and HSB2b, respectively (Supplemental Fig. S2F). The highest shared identity for the OD is also observed for HSFB2a and HSFB2b: 43.48%. SCZ, on the other hand, shares 36.96% and 32.61% identity with HSFB1 and HSFB2b, respectively (Supplemental Fig. S2G). Therefore, despite being the 2 domains common to all HSFs, both the DBD and the OD may still carry sufficient variation to justify the functional divergence between SCZ and the stress-related HSFBs.

During the course of this research, we generated an additional mutant for *SCZ* by means of CRISPR/Cas9 (hereafter referred to as *scz^CR^*). The *scz^CR^* allele, which is phenotypically identical to previously described *scz* alleles, was used specifically for the domain-swapping studies described below. *scz^CR^* contains a thymidine insertion in the first exon of the *SCZ* gene, creating a premature stop codon (Supplemental Fig. S1, orange asterisk) that leads to the production of a 68-amino acid-long peptide containing ∼38% of the DBD and no other SCZ domain. Unlike the scz-2 allele, the lack of any (complete) domains residing in the small putative translated protein in the *scz^CR^* mutant avoids interference due to protein–DNA or protein–protein interaction.

**Figure 4.**
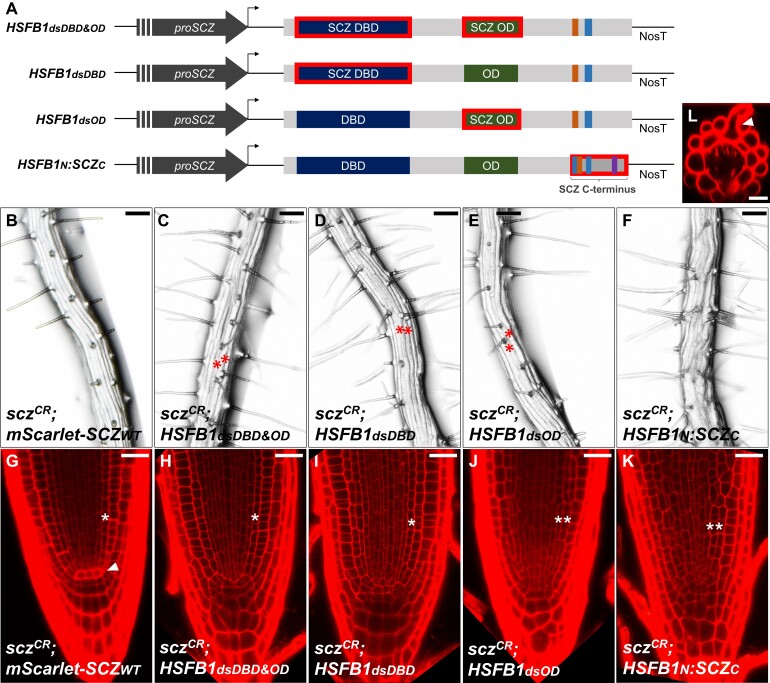
Complementation tests of *scz^CR^* by HSFB1 versions containing SCZ domains. **A)** Schematic representation of the constructs containing *proSCZ* driving *HSFB1-SCZ* hybrid versions. Indicated are DBD in dark blue, OD in green, RD in orange, NLS in light blue, and NES/EAR in purple. The domain outlined in red was replaced by the corresponding SCZ domain. **B to K)** Bright field images of the mature root zone and corresponding confocal images of *scz^CR^* root tips complemented with *proSCZ::mScarlet-SCZ_WT_***B**, **G)**, *proSCZ::HSFB1_dsDBD&OD_***C**, **H)**, *proSCZ::HSFB1_dsDBD_***D**, **I)**, *proSCZ::HSFB1_dsOD_***E**, **J)**, and *proSCZ::HSFB1_N_:SCZ_C_***F**, **K)**. **L)** Digital cross-section of *scz^CR^;proSCZ::HSFB1_dsOD_* showing subepidermal root hair emerging between 2 epidermal cells (white arrowhead). For confocal imaging, fresh 5dag roots were stained with PI. Red asterisks mark adjacent root hair files. White asterisks mark either single or double endodermis. Scale bar = 100 *μ*m **B to F)** or 25 *μ*m **G to L)**.

To point out the specific domain that differentiates SCZ from the other HSFBs tested for complementation, we generated HSFB1 variants with domains switched (ds) by those from SCZ, creating HSFB1_dsDBD&OD_, HSFB1_dsDBD_, HSFB1_dsOD_, and HSFB1N:SCZC. The coding sequence of these hybrid proteins was again placed under the control of *proSCZ* and used to test for *scz^CR^* mutant complementation (Fig. 4A). As a control, the complementation of *scz^CR^* with *mScarlet-SCZ_WT_* resulted in the full rescue of mutant root phenotypes (Fig. 4, B to G). From the 4 swap versions generated, the only one that did not result in any level of rescue was HSFB1N:SCZC, in which the C-terminal region of HSFB1 was replaced by that of SCZ (Fig. 4, F and K). The other swapped versions, HSFB1_dsDBD&OD_, HSFB1_dsDBD_, and HSFB1_dsOD_, resulted in morphological improvement of the root epidermal cell file tractability (Fig. 4, C to E), segregating again as a dominant trait. However, similar to what was observed in the *scz-2* plants transformed with the full HSFB1 or HSFB2b versions, the root hair pattern is not rescued from the presence of adjacent hair cell files, and, additionally, in HSFB1_dsOD_, subepidermal root hairs were occasionally observed (Figs. 3, C, D, and H, and 4, C to E and L). Nevertheless, in *scz^CR^*;*HSFB1_dsDBD&OD_* and *scz^CR^*;*HSFB1_dsDBD_* lines, 44% and 50%, respectively, of the roots that display improved epidermis tractability also restored the endodermis to a single layer (Fig. 4, H and I; Supplemental Table S1). This is in contrast to *scz^CR^;HSFB1_dsOD_*, *scz-2;proSCZ::HSFB1*, and *scz-2;proSCZ::HSFB2b* lines (Supplemental Table S1). In addition, 100% of *scz^CR^;HSFB1_dsDBD&OD_* and *scz^CR^;HSFB1_dsDBD_* roots that display improved epidermis tractability exhibited a closed meristem phenotype, and the majority of roots display a restored columella pattern (Fig. 4, H and I; Supplemental Table S1). On the other hand, among the *scz^CR^;HSFB1_dsOD_* roots that display improved epidermis tractability, only ∼30% presented a rescued columella and closed meristem phenotype (Supplemental Table S1). Thus, even though the complementation by *HSFB1_dsDBD&OD_* and *HSFB1_dsDBD_* expression is not as complete as observed for *scz^CR^*;*mScarlet-SCZWT*, considering that up to 50% of the roots display single endodermis, the overall level of rescue is superior to what was observed in the complementation with native HSFB1 or HSFB2b coding sequences (Fig. 3, F and G; Supplemental Table S1). These results indicate that the DBD, despite being the most conserved domain between SCZ and HSFB1 (Supplemental Fig. S2), is predominantly responsible for differentiating SCZ from other HSFBs when it comes to its specific role in root patterning.

## Discussion

Prior studies in animals and plants have demonstrated the requirement of the OD for the formation of homotrimers and heterooligomers between HSFs (Sorger and Nelson 1989; Hubel et al. 1995; Orosz et al. 1996; Scharf et al. 1998; Baniwal et al. 2007; Chan-Schaminet et al. 2009; Sandqvist et al. 2009); the role of the DBD in binding to HSEs at the promoter of transcriptional targets (Cicero et al. 2001; Guo et al. 2008; Chan-Schaminet et al. 2009; Feng et al. 2021; Schmauder et al. 2022); and the importance of the subcellular localization signals for the traffic of HSFs between nucleus and cytosol (Heerklotz et al. 2001; Kotak et al. 2004; Zhu et al. 2012). Similar (domain) studies, including SCZ, seem particularly relevant, given that SCZ appears to be mainly involved in developmental rather than stress-related processes (Mylona et al. 2002; Pernas et al. 2010; ten Hove et al. 2010; Begum et al. 2013; Olmo et al. 2020). Here, we have successfully combined site-directed mutagenesis with transient expression and genetic complementation experiments to address the importance of conserved domains and motifs for SCZ subcellular localization and function.

The abolishment of SCZ function due to mutations in the DBD or OD is not surprising since these domains are typical of all HSFs, even across kingdoms. These domains confer DNA-binding capacity and/or the formation of homo/heterotrimers, which are essential for HSF function (Nover et al. 2001). Contrary to expectation, separate mutations in the C-terminal NLS, RD, or NES/EAR motif only were able to completely rescue the *scz-2* root defects. This expectation arises from the fact that the *scz-2* mutant allele has a 1-base pair deletion in the 3′-end of the *SCZ* coding sequence, just after the first part of the encoded bipartite NLS and at the beginning of the RD. This mutation leads to a frameshift in the ORF and a consequent phenotype that is identical to described alleles with 5′-end mutations, indicating total loss of protein function (ten Hove et al. 2010). It was questioned, therefore, whether mutations in either the NLS or NES/EAR domain are sufficient to confine SCZ into distinct subcellular compartments. Indeed, whereas SCZ_mNES/EAR_ was exclusively nuclear, SCZ_mNLS_ was present in the cytosol but not excluded from the nucleus. The results suggest that the NLS is not the only signal responsible for the nuclear enrichment of SCZ. Unusual NLS have been identified in many proteins and may even be formed only upon protein dimerization (Lu et al. 2021). A strong candidate for a redundant NLS is the DBD since mutations in this domain led to enhanced cytosolic mScarlet-SCZ_mDBD_ accumulation in *N. benthamiana*. Importantly, in Arabidopsis roots, accumulation of both mScarlet-SCZ_mNES/EAR_ and mScarlet-SCZ_mNLS_ is observed in the domain that fully overlaps with the previously reported accumulation pattern of *SCZ* mRNA (ten Hove et al. 2010). This indicates that SCZ protein is not moving from its cortical expression domain to outer tissue layers to exert its noncell-autonomous role in segregating the epidermis, lateral root cap, and columella identities. Instead, at least 1 SCZ transcriptional target or its metabolic product may be moving to the outer layers.

Accumulation of mScarlet-SCZWT was detected only when expressed from the *35S* promoter in transient expression assays in *N .benthamiana* leaves and then the protein localized to the nucleus. In Arabidopsis, SCZWT fused to the bright mScarlet fluorescent protein remained undetectable but fully rescued *scz-2*, proving its functionality. The fact that mScarlet-SCZ_mNES/EAR_ is detectable in Arabidopsis root meristem cells could mean that the mutation in the NES traps the protein in the nucleus, consequently increasing its nuclear concentration beyond detection levels. Similar nuclear enrichment was observed either when the nuclear export was compromised by the addition of leptomycin B or when the NES from 4 tomato (*Solanum lycopersicum*) HSFs (SlHSFA1a, SlHSFA1b, SlHSFA2, and SlHSFA8) was mutated (Heerklotz et al. 2001; Kotak et al. 2004). However, mScarlet-SCZ_mNLS_ is also detectable in Arabidopsis, which would theoretically be less present in the nucleus. Thus, our results do not clarify whether the NES is involved in nuclear export. An alternative hypothesis is that these mutations at the C-terminus enhance protein stability, increasing its concentration and visibility.

Unexpected was the ability of SCZ_mRD_ to rescue *scz-2* defects, suggesting that this RD domain does not confer transcriptional repression. Indeed, a previously reported transient transcription activity assay in *N. benthamiana* failed to confirm SCZ-repressive activity (Ikeda et al. 2011; Zhu et al. 2012). To account for this, we considered that the putative NES overlaps with an also putative EAR-repressive motif (Ohta et al. 2001; ten Hove et al. 2010). In line with this, the EAR-like domain from the WUSCHEL transcription factor has been previously reported to function both as a repressive motif and a NES (Rodriguez et al. 2016; Plong et al. 2021). Since no differences in subcellular localization were observed between mScarlet-SCZWT and mScarlet-SCZ_mNES/EAR_ when expressed in *N. benthamiana*, we argue that the putative NES/EAR may act as an EAR-repressive motif instead. Indeed, a SCZ_mRD + mNES/EAR_ variant failed to rescue *scz-2* phenotypes, suggesting that 1 repressive motif can compensate for the loss of the other. In addition, SCZ C-terminus exchange with the repression domain from HSFB1 (SCZN:HSFB1C) or addition of the SRDX-repressive motif to the C-terminus of SCZ (SCZ:SRDX) allowed the complete rescue of *scz-2* defects. Conversely, the addition of the coding sequence of the VP16 strong transcriptional activator domain to the *SCZ* C-terminus (*SCZ + VP16*), expressed from *proSCZ* in Col-0 (WT) plants, conferred *scz-*like phenotypes. The latter result can be explained by SCZ:VP16 ectopically inducing target genes, which is equivalent to compromising SCZ-repressive function. Together, these results reinforce the idea that SCZ acts as a transcriptional repressor, that its repressive activity depends on the RD and NES/EAR domains, and that its repressive ability is essential for its role in root patterning.

Functional redundancy has been reported for HSFAs (HSFA1a, HSFA1b, HSFA1d, and HSFA1e) and HSFBs (HSB1 and HSFB2b) through the analysis of double, triple, and quadruple mutants in Arabidopsis (Ikeda et al. 2011; Liu et al. 2011). Interestingly, *proSCZ::HSFB1* and *proSCZ::HSFB2b* in *scz-2* visibly improved root epidermis and meristem morphology aspects but failed to fully complement mutant defects. This indicates that other *HSFBs*, when expressed in the *SCZ* domain, have the ability to regulate the expression of at least a subset of SCZ targets. This is remarkable since other HSFBs share, at best, ∼40% overall amino acid identity with the SCZ sequence. Nevertheless, the results made clear that the expression domain is not the only factor differentiating SCZ from other HSFBs, but that there are also functionally relevant differences between their amino acid sequences. Indeed, phenotypic rescue by HSFB1_dsDBD&OD_ and HSFB1_dsDBD_ was superior to the rescue by *proSCZ::HSFB1* or *proSCZ::HSFB2b*. This indicates that despite being the most conserved domain across HSFs, the DBD still contains sufficient differences allowing it to be the major domain responsible for the functional divergence between SCZ and the stress-related HSFBs. Importantly, like SCZ, HSFB2a is also known for playing a developmental role, as evidenced by its implication in gametophyte development (Wunderlich et al. 2014). This way, future experiments can help elucidate whether HSFB2a and SCZ share characteristics that confer a developmental role rather than involvement in stress response. Nevertheless, unlike complementation with mScarlet-SCZWT, HSFB1_dsDBD&OD_ and HSFB1_dsDBD_ variants were not able to fully rescue the epidermal defects, indicating that there is still some residual specificity promoted by regions outside of the DBD and OD. Such regions may be important for recruitment to specific regulatory domains on the genome.

In summary, the results presented here clarify the importance of conserved domains and motifs for SCZ function in root patterning. Our data strongly suggest a role for SCZ as a transcriptional repressor, which seems to be essential to its function and relies on 2 motifs. Additionally, the data shed light on the functional divergence between SCZ and 2 stress-related HSFs from the same clade. However, it remains unclear how SCZ exerts its nonautonomous role. Our results indicate that the protein is not moving to the epidermis, lateral root cap, or columella. Therefore, this study justifies future analyses of the downstream regulated genes that mediate the (nonautonomous) effects of SCZ and clarifies the difference between stress and the developmental function of HSFB family members.

## Materials and methods

### Plant material and growth conditions

Arabidopsis (*A. thaliana*) Col-0 (WT), *scz-*2 (ten Hove et al. 2010), *scz^CR^*, and transgenic seeds were gas sterilized for 2 h as described in Lindsey et al. (2017), resuspended in sterile 0.1% agarose (*w*/*v*), and stratified at 4 °C for 72 h. The seeds were plated on one-half strength “Murashige & Skoog Medium with vitamins” growth media, pH 5.7, supplemented with 0.8% plant agar (*w*/*v*), 1% sucrose (*w*/*v*), and 0.05% MES monohydrate (*w*/*v*) (all from Duchefa Biochemie). Plates were positioned almost vertically, and seedlings were grown at 21 °C in a light/dark regime of 16 h/8 h. Seedling age was counted from root protrusion onwards as “days after germination” (dag).

### Construct design and cloning

*SCZ* (mutant) variants and other constructs were generated using the Golden Gate cloning method with the MoClo Toolkit and Plant parts (Addgene Kits # 1000000044 and #1000000047) (Engler et al. 2014; Engler and Marillonnet 2014) and appropriate sets of primers (Supplemental Table S2). Primers were designed to introduce nonsynonymous mutations in *SCZ* conserved domains/motifs and to introduce synonymous mutations to remove BsaI and BpiI restriction sites that interfere in the cloning process. For each variant, fragments were amplified by PCR using high-fidelity Phusion Taq polymerase, purified and combined into level 0 acceptors. The level 0 modules containing either *proSCZ* (pICH41233-proSCZ) or *pro35S* (pICH41388, for agroinfiltration), the 5′-UTR omega(TMV) (pAGT707), the mScarlet (pAGM1276-mScarlet), the *SCZ* or *HSFB* variant assembled into pICH41308 (with STOP codon) or pAGM1287 (without STOP codon), the C-terminus tag (when appropriate) pAGM1301-SRDX or pAGM1301-VP16, and the Nopaline Synthase terminator (pICH41421-NosT) were combined into the level 1 acceptor pICH47742 to generate transcriptional units. Finally, the obtained pICH47742 level 1 module was combined with pICH47732-BAR (*proNOS::BAR* resistance cassette) and the pICH41744 end-linker into the level 2 acceptor pICSL4723. Cloning was performed in *Escherichia coli* DH5α, and confirmed constructs were subsequently transformed into *Agrobacterium tumefaciens* strain C58C1.pMP90 (Koncz and Schell 1986) for plant transformation. The final level 2 constructs containing *proSCZ*-driven genes were transformed into Col-0, *scz-2*, and/or *scz^CR^* by means of the floral dip method (Clough and Bent 1998). Transgenic lines were obtained for each construct based on selection for resistance against phosphinothricin (PPT) mediated by BAR gene expression.

CRISPR/Cas9-mediated mutagenesis was used to generate the *SCZ* knockout allele *scz^CR^* (in Col-0). The construct *pICSL4723-FASTR-RPS5a::aCas9-SCZsgRNA1-SCZsgRNA10* was generated using Golden Gate cloning (Engler and Marillonnet 2014), as described below. CRISPR-P2.0 was used to design spacer sequences 5′-ATGATGGTCGAGAATAGCTA and 5′-AGATCACGAGCAAACTCCGG. These sequences and their reverse complement were ordered as oligos with a 4-nucleotide extension of ATTG at the 5′-end of the sense and AAAC at the 5′-end of the reverse complement. Oligos were annealed, and the overhangs allowed direct Golden Gate cloning behind the Arabidopsis RNA pol III promoter *AtU6-26* into a predesigned level 0 vector *pU6_26-RFP-sgRNAopt* using BsmBI restriction-ligation, thereby replacing the lac promoter-driven RFP gene. Subsequently, the *AtU6-26*-driven sgRNA cassettes were cloned into lvl1 vectors *pICH47751* and *pICH47761* by BsaI restriction-ligation, to create *pICH47751-pAtU6_26-SCZsgRNA1* and *pICH47761-pAtU6_26-SCZsgRNA10*. Subsequently, level 1 vectors harboring sgRNAs were combined with *pICH47732-FAST_R* (red seed selection), *pICH47742-RPS5a::aCas9*, and the end-linker *pICH41780* into the level 2 binary vector *pICSL4723* using BpiI restriction-ligation. Plasmid *pICH47732-FAST_R* was generated by Golden Gate cloning of the *FAST-R* selection cassette from *pICSL7008* (monomeric tagRFP from *Entacmaea quadricolor* driven by the Arabidopsis *OLEOCIN 1* promoter *AtOLE1*) into *pICH47732*. Plasmid *pICH47742-RPS5a::aCas9* was generated by assembling *pICH41233-RPS5a*, *pICH41308-aCas9*, and *pICH41421* (*T_NOS_*) into *pICH47742*. The *RPS5a* promoter was amplified from *A. thaliana* genomic DNA using pRPS5AF-BpiGGAG and pRPS5AR-BpiTACT, followed by Golden Gate cloning into *pICH41233*. A plasmid harboring the Arabidopsis codon-optimized aCas9 was kindly provided by the Puchta lab (Fauser et al. 2014) and amplified using primers aCas9F-BpiAATG and aCas9R-BpiGCTT followed by Golden Gate cloning into *pICH41308*. Plants were transformed by floral dip (Clough and Bent 1998), and T1 transgenic seeds were selected under a fluorescence binocular (Leica MZ16F). Inflorescences of T1 CRISPR/Cas9 mutagenized plants were genotyped for induced mutation by PCR using primers SCZ-CRISPRF and SCZ-CRISPRR, followed by sequencing. Seeds of mutant plants were selected for the absence of the CRISPR/Cas construct, followed by genotyping for homozygosity of the mutation.

### Microscopy and phenotyping

Pictures of the root mature zone were taken with a stereoscopic microscope Nikon SMZ745T, imaged directly from the agar plates. For confocal pictures of live seedling roots, the roots were mounted on microscopy slides with 5 *μ*g/mL propidium iodide (PI), covered with a coverslip and immediately imaged. For confocal imaging of mScarlet fluorescence, roots were treated following the ClearSee method and either observed without any stain or stained with Direct Yellow 96 (Sigma) as described in Ursache et al. (2018). Roots were mounted on microscopy slides with ClearSee solution and covered with a coverslip immediately prior to imaging. For imaging of agroinfiltrated *N. benthamiana* leaves, fresh samples were collected and mounted with water on microscopy slides and covered with a coverslip. The Zeiss LSM 710 confocal microscopy was used for imaging, with the 40× water immersion objective. For imaging PI-stained roots, the 514 nm laser was used for excitation. For imaging mScarlet and cell walls stained with Direct Yellow 96, the 488 and 543 nm lasers were used for excitation. For imaging mScarlet fluorescence in *N. benthamiana* leaves, the 543 nm laser was used. For collection, the ranges 599 to 719 (PI), 590 to 631 (mScarlet), and 491 to 539 (Direct Yellow) were defined.

Considering that phenotypic analyses were performed in second-generation segregating lines and that the *scz* mutation is recessive, ∼75% of the roots were expected to show phenotypic rescue to WT in complementing lines. This way, mScarlet-tagged SCZ variant lines exhibiting restoration of QC morphology, presence of a single endodermis, and absence of subepidermal root hairs in ∼75% of the roots were considered “fully complementing lines.” Transgenic lines that appeared to restore some or none of these features were considered “noncomplementing” lines (Supplemental Table S3). Two “fully complementing lines” were selected for each construct and used for further analysis. For the mScarlet-SCZ_mOD_ construct not rendering *scz-2* phenotypic rescue nor fluorescence signal, more independent lines were analyzed compared to the other constructs that did either complement the *scz-2* phenotype to WT or displayed fluorescence. Phenotypic rescue of *scz-2* and *scz^CR^* plants that were transformed with *proSCZ::HSFB1*, *proSCZ::HSFB2b*, and *proSCZ::HSFB1_DOMAINSWAPS_* (Supplemental Table S1) was scored for columella phenotype (WT or mutant), meristem organization (open or closed), and number of endodermal layers (single or double). This was done for the root meristems of the best complementing line for each construct and only for those roots displaying root epidermal pattern improvement. The best complementing line for each construct was defined as the line with the highest proportion of roots displaying WT columella, closed meristem organization, and single endodermis out of a total of 23 *scz-2;proSCZ::HSFB1* T2 lines, 24 *scz-2;proSCZ::HSFB2b* T2 lines, 13 *scz^CR^;proSCZ::HSFB1_dsDBD&OD_* T2 lines, 15 *scz^CR^;proSCZ::HSFB1N:SCZC* T2 lines, 15 *scz^CR^;proSCZ::HSFB1_dsDBD_* T2 lines, and 19 *scz^CR^;proSCZ::HSFB1_dsOD_* T2 lines.

### Agroinfiltration of *N. benthamiana* leaves

Agroinfiltration was performed largely according to Diaz-Trivino et al. (2017). In brief: *A. tumefaciens* colonies carrying the *pICSL4723-BAR-35S::mScarlet-SCZ_VERSION_* plasmids were inoculated in 5 mL LB culture (with the appropriated antibiotics) and grown at 28 °C for ∼48 h. The same was done to *Agrobacterium* carrying the P19 silencing suppressor plasmid. The *Agrobacterium* cultures were then subcultured (1:100 ratio, *v*/*v*) into new 5 mL LB medium with 10 mM MES (pH 5.6) and 40 *μ* M acetosyringone. Bacteria were grown at 28 °C until an OD_600_ of ∼3.0 and then gently pelleted (3,200 g, 10 min). The pellets were resuspended in 10 mM MgCl_2_ to an OD_600_ = 0.5, and acetosyringone was added to a final concentration of 200 *μ* M. The bacteria were kept at room temperature for at least 3 h without shaking. The *Agrobacterium* cultures containing the *pICSL4723-BAR-35S::mScarletSCZ_VERSION_* and the p19-helper and 10 mM MgCl2 were mixed in a *v*/*v* proportion of 3:1 with a 5-mL syringe, and the mixed solution was infiltrated in young leaves of 14-d-old *N. benthamiana* plants. Three days after infiltration, the leaves were observed under the microscope.

### Accession numbers

Plant genes mentioned in this article can be found under the following accession numbers: *SCZ* (*HSFB4*, *AT1G46264*); *HSFA1s* being *HSFA1A* (*AT4G17750*), *HSFA1B* (*AT5G16820*), *HSFA1D* (*AT1G32330*), and *HSFA1E* (*AT3G02990*); *HSFA2* (*AT2G26150*), *HSFB1* (*AT4G36990*), *HSFB2A* (*AT5G62020*), *HSFB2B* (*AT4G11660*), *HSFB3* (*AT2G41690*), *WUSCHEL* (*AT2G17950*), *SMB* (*AT1G79580*), *SlHSFA1A* (*Sl08G005170*), *SlHSFA1B* (*Sl03G097120*), *SlHSFA2* (*Sl08G062960*), *SlHSFA8* (*Sl09G059520*), and *OLE1* (*AT4G25140*).

## Supplementary Material

kiad456_Supplementary_Data

## Data Availability

All study data are included in the article and/or supplementary materials. The Arabidopsis lines and plasmids used in this study will be shared upon request.

## Supplemental data

The following materials are available in the online version of this article.

**Supplemental Figure S1.** Amino acid alignment of the HSFB family members.

**Supplemental Figure S2.** Amino acid alignment of the conserved domains and motifs of Arabidopsis HSFB family members.

**Supplemental Figure S3.** Complementation of the subepidermal root hair phenotype in *scz-2;proSCZ::mScarlet-SCZ_WT_*.

**Supplemental Figure S4.** *proSCZ::mScarlet-SCZ _+ VP16_* promotes root hair defects in Col-0.

**Supplemental Figure S5.** Expression of *HSFB* members in roots.

**Supplemental Figure S6.** Representative example of closed and open meristem organization.

**Supplemental Table S1.** Frequency of phenotypical rescue of *scz-2* and *scz^CR^* complemented with *proSCZ::HSFB1*, *proSCZ::HSFB2b*, and *proSCZ::HSFB1_DOMAINSWAPS_*.

**Supplemental Table S2.** Primers and cloning scheme.

**Supplemental Table S3.** Frequency of phenotypic rescue of *scz-2* and *scz^CR^* complemented with mScarlet-tagged SCZ variants.

## Dive Curated Terms

The following phenotypic, genotypic, and functional terms are of significance to the work described in this paper:

HSFB4 Gramene: AT1G46264
HSFB4 Araport: AT1G46264

AT1G46264 Gramene: AT1G46264
AT1G46264 Araport: AT1G46264

AT1G32330 Gramene: AT1G32330
AT1G32330 Araport: AT1G32330

AT3G02990 Gramene: AT3G02990
AT3G02990 Araport: AT3G02990

AT4G11660 Gramene: AT4G11660
AT4G11660 Araport: AT4G11660

AT4G17750 Gramene: AT4G17750
AT4G17750 Araport: AT4G17750

AT5G16820 Gramene: AT5G16820
AT5G16820 Araport: AT5G16820

AT5G62020 Gramene: AT5G62020
AT5G62020 Araport: AT5G62020

GL2 Gramene: AT1G79840
GL2 Araport: AT1G79840

MES CHEBI: CHEBI:39010

HSFA1a Gramene: At1g32330
HSFA1a Araport: At1g32330
