# Supplementary material for: SCHIZORIZA domain–function analysis identifies requirements for its specific role in cell fate segregation
Source: Plant Physiol. 2023 Aug 16;193(3):1866–79. doi: 10.1093/plphys/kiad456 (PMC10602604; doi:10.1093/plphys/kiad456)
Supplement: kiad456_Supplementary_Data [file kiad456_supplementary_data.pdf]

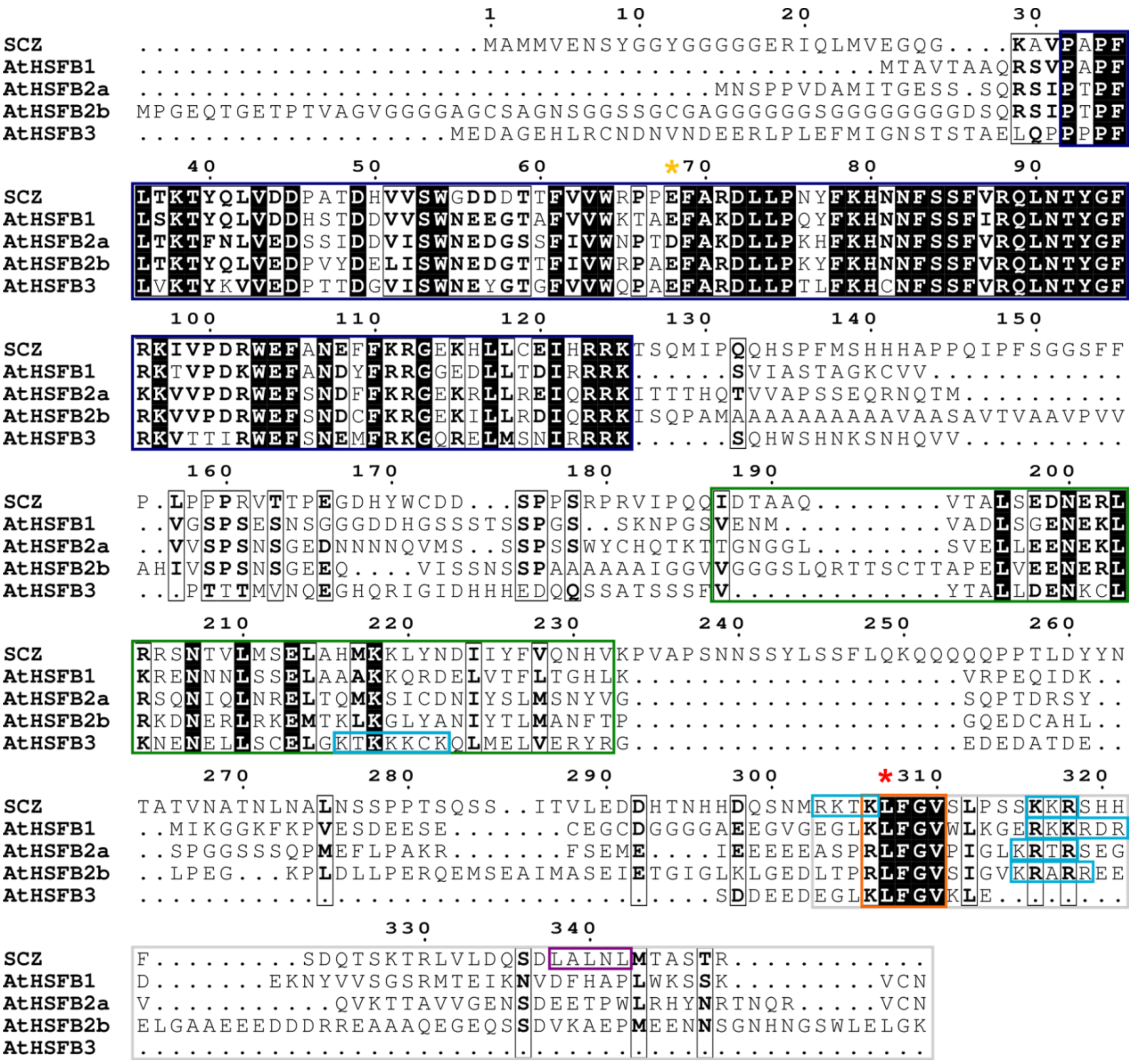

Supplemental Figure S1. Amino acid alignment of the HSFB family members. Predicted amino acid sequences were aligned by the Muscle method at the EMBL-EBI browser and plotted with ESPript (Edgar, 2004, Robert and Gouet, 2014, Madeira et al., 2022). Conserved domains/motifs are marked by coloured boxes: dark blue = DBD; green = OD; light blue = NLS; orange = RD; and purple = NES/EAR-motif. Bold amino acids indicate physicochemical similarity > 0.7 across members, whereas white font amino acids on black background indicates strict identity across all members. Orange asterisk indicates premature STOP codon created by *scz*^{CR} mutation. Red asterisk indicates the first amino acid affected by *scz-2* mutation.

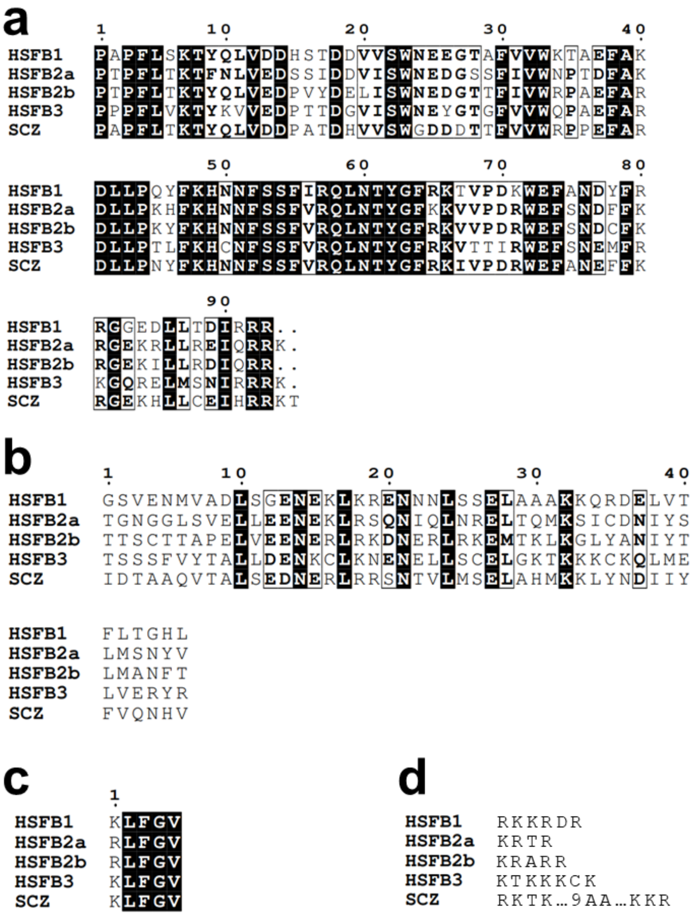

e

%identity	SCZ	HSFB2a	HSFB2b	HSFB3	HSFB1
SCZ	100				
HSFB2a	36.46	100			
HSFB2b	37.22	49.66	100		
HSFB3	38.3	37.78	37.08	100	
HSFB1	39.34	39.19	39.35	43.26	100

f

%identity	HSFB3	HSFB1	SCZ	HSFB2a	HSFB2b
HSFB3	100				
HSFB1	64.52	100			
SCZ	64.89	72.04	100		
HSFB2a	62.77	66.67	70.21	100	
HSFB2b	68.82	70.97	77.42	80.65	100

g

%identity	HSFB3	HSFB2a	HSFB2b	SCZ	HSFB1
HSFB3	100				
HSFB2a	30.43	100			
HSFB2b	28.26	43.48	100		
SCZ	26.09	32.61	32.61	100	
HSFB1	32.61	26.09	21.74	36.96	100

Supplemental Figure S2. Amino acid alignment of the conserved domains and motifs of Arabidopsis HSFB family members. (a-c) Alignment of the DBD **(a)**, OD **(b)** and RD **(c)**. **(d)** Sequences of the predicted NLS of HSFBs. Due the low similarity, these sequences were not aligned. **(e-g)** 'Percent Identity Matrixes' denoting percentage of identical amino acid residues in relation to the length of the alignment between the full HSFB protein sequences **(e)**, their DBD **(f)** and their OD **(g)**. In red, identity between SCZ and other HSFBs. To generate the protein alignments and identity matrixes, predicted amino acid sequences were aligned by the Muscle method at the EMBL-EBI browser and plotted with ESPript (Edgar, 2004, Robert and Gouet, 2014, Madeira et al., 2022). Bold amino acids indicate physicochemical similarity > 0.7 across members, whereas white font amino acids on black background indicates strict identity across all members.

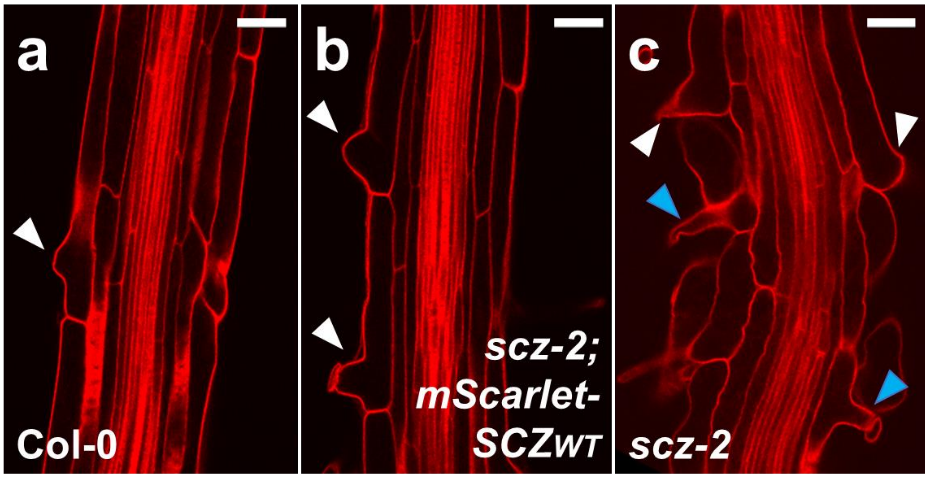

Supplemental Figure S3. Complementation of the subepidermal root hair phenotype in *scz-2;proSCZ::mScarlet-SCZ$_{WT}$*. Imaging of the mature zone of the root of Col-0 **(a)**, *scz-2;proSCZ::mScarlet-SCZ$_{WT}$* **(b)** and *scz-2* **(c)** by confocal laser microscopy. The phenotypic restoration observed in **(b)** is representative for all complementing lines. White arrowheads indicate epidermal root hairs, blue arrowheads indicate subepidermal root hairs in *scz-2* roots. Scalebar in all panels = 25 µm

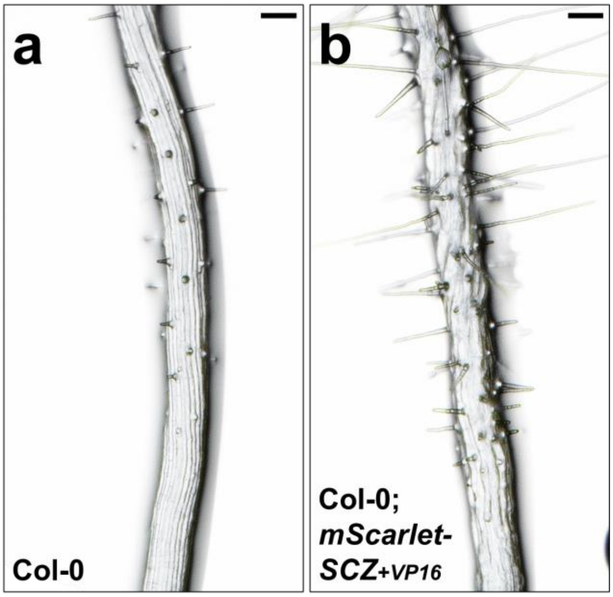

Supplemental Figure S4. *proSCZ::mScarlet-SCZ$_{+VP16}$* promotes root hair defects in Col-0. Bright field images of the mature root zone of **(a)** Col-0 (WT) and; **(b)** Col-0;*proSCZ::mScarlet-SCZ$_{+VP16}$* displaying *scz-2*-like phenotype. Scale bar in both panels = 100 µm.

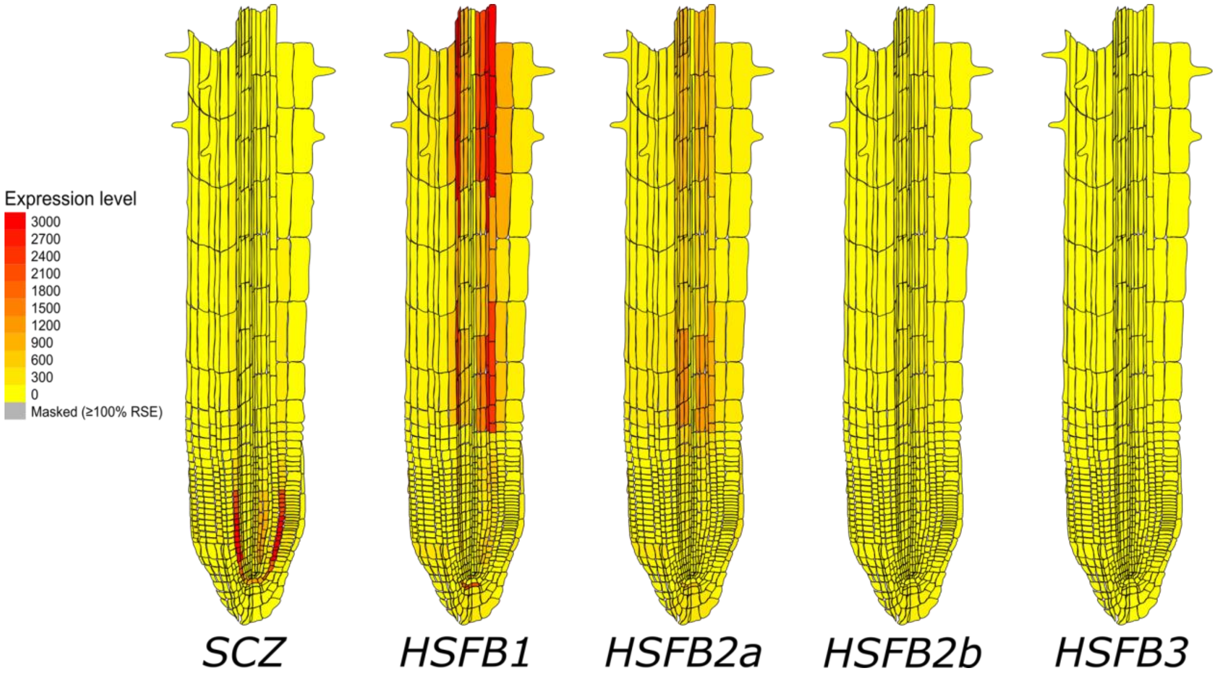

Supplemental Figure S5. Expression of *HSFB* members in roots. Schematic representation of *SCZ* and its family members mRNA expression levels in the Arabidopsis root according to (Brady et al., 2007) visualized by the *Arabidopsis* eFP Browser (Winter et al., 2007). A cut-off is set at 3000 arbitrary units.

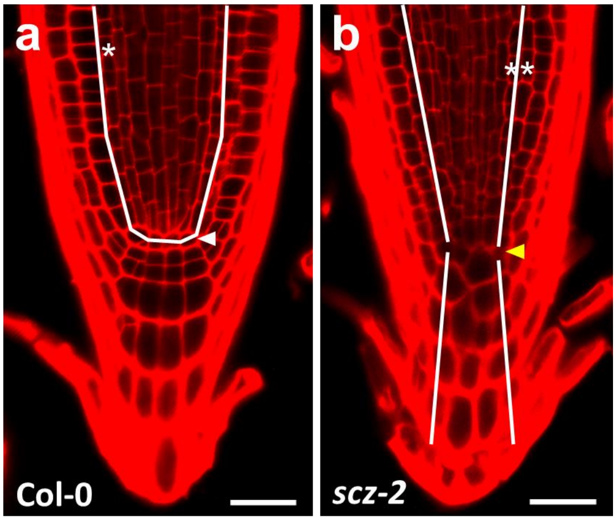

Supplemental Figure S6. Representative example of closed and open meristem organization. (a) Col-0 root exhibiting a closed meristem organization, with a clear separation between the longitudinal stretched vascular cells and columella by the fusion of the ground tissue through horizontal QC cells, the U-domain (white line). The columella root cap shows well-organized cell columns. **(b)** Mutant *scz-2* root displaying an “open meristem” like organization, lacking the U-domain due to absence of QC, and therefore it appears as if files of cells from the meristem continue into the columella (white lines). The mutant columella, is disorganized and lacks clear cell columns. Asterisks indicate endodermis, white arrowhead indicates QC, yellow arrowhead indicates absence of QC. Scale bar in both panels = 25 µm.

Supplemental Table S1. Frequency of phenotypical rescue of *scz-2* and *scz*^{CR} complemented with *proSCZ::HSFB1*, *proSCZ::HSFB2b* and *proSCZ::HSFB1*$_{DOMAINSWAPS}$. The table depicts the frequency of phenotypic rescue of the best complementing line for each construct. The root meristem was scored for columella phenotype (WT or mutant), meristem organization (open or closed) and number of endodermal layers (single or double). Considering that the analyses were performed in second generation segregating lines, only roots displaying root (epidermal) pattern improvement were used for scoring (see Figures 3 and 4). n is number of roots for the particular line.

Genotype	WT columella	Single endodermis	Closed meristem	*n*
scz-2;pSCZ::HSFB1 (T2-6)	0.80	0.00	1.00	10
scz-2;pSCZ::HSFB2b (T2-12)	0.75	0.00	0.75	8
scz^{CR}*;HSFB1*$_{dsDBD\&OD}$ (T2-12)	0.67	0.44	1.00	18
scz^{CR}*;HSFB1*$_{dsDBD}$ (T2-15)	0.67	0.50	1.00	12
scz^{CR}*;HSFB1*$_{dsOD}$ (T2-12)	0.29	0.00	0.29	14

Supplemental Table S2. Primers and cloning scheme.

Primer name	Primer sequence (5'-3')	Purpose	Level 0 Assembly
pSCZ-GGF1	TGTGAAGACAAGGAGGCAAGCAACAACATTATACCAA	Amplify *proSCZ* and remove 2 BpiI restriction sites. Three PCR reactions using primer pairs with the same number. Use Col-0 genomic DNA as template.	Assemble 3 fragments into pICH41233
pSCZ-GGR1	TGTGAAGACAATCTTCCTAGCAGTAAATAAGAAAAA		
pSCZ-GGF2	TGTGAAGACAAAAGAGATTTAGATTTAGAGATATTAGAAGAATAATTTG		
pSCZ-GGR2	TGTGAAGACAATAGACTTGTGATATCTACTTTTGCTTT		
pSCZ-GGF3	TGTGAAGACAATCTACCAATTTCATATATATCAGGAGAAAAA		
pSCZ-GGR3	TGTGAAGACAAAGTATTCGAGAAAGAGAGAGACAGAGAG		
SCZ-GGF1	TGTGAAGACAAAATGGCGATGATGGTCGAG	Amplify SCZ CDS *(*SCZc*)* and remove 2 BpiI restriction sites. Three PCR reactions using primer pairs with the same number. Use Col-0 cDNA as template.	Assemble 3 fragments into pICH41308
SCZ-GGR1	TGTGAAGACAACTCTTCTGGAGTGGTGACACG		
SCZ-GGF2	TGTGAAGACAAAGAGGACCATTACTGGTGCGACGA		
SCZ-GGR2	TGTGAAGACAATATCTTCACTCAATGCCGTTACTT		
SCZ-GGF3	TGTGAAGACAAGATAACGAGAGATTACGACGAAGC		
SCZ-GGR3	TGTGAAGACAAAAGCTTAACGTGTAGAAGCAGTCATGAGA		
SCZg-F1	TGTGAAGACAAAATGGCGATGATGGTCGAG	Amplify genomic *SCZ* with STOP codon (SCZg) and remove 3 BpiI restriction sites to generate SCZWT. Four PCR reactions using primer pairs with the same number. Use Col-0 genomic DNA as template.	Assemble 4 fragments into pICH41308
SCZg-R1	TGTGAAGACAATAGAAGCAGACAAAAAAACTCAAAAAC		
SCZg-F2	TGTGAAGACAATCTACAAGAAATCTGATGTTATGATCTT		
SCZg-R2	TGTGAAGACAACTCTTCTGGAGTGGTGACACG		
SCZg-F3	TGTGAAGACAAAGAGGACCATTACTGGTGCGACGA		
SCZg-R3	TGTGAAGACAATATCTTCACTCAATGCCGTTACTT		
SCZg-F4	TGTGAAGACAAGATAACGAGAGATTACGACGAAGC		
SCZg-R4	TGTGAAGACAAAAGCTTAACGTGTAGAAGCAGTCATGAGA		
HSFB1-F	TGTGAAGACAAAATGACGGCTGTGACGGCG	Amplify HSFB1 CDS. Use Col-0 cDNA as template.	Assemble into pICH41308
HSFB1-R	TGTGAAGACAAAAGCTTAGTTGCAGACTTTGCTGCTT		
HSFB2b-F	TGTGAAGACAAAATGCCGGGGGAACAAAC	Amplify HSFB2b CDS. Use Col-0 cDNA as template.	Assemble into pICH41309
HSFB2b-R	TGTGAAGACAAAAGCTCATTTTCCGAGTTCAAGCC		

Supplemental Table S2 continued

SCZg_N-R1	TGTGAAGACAATCCATATTACTCTGATCATGATGATTAGTGTG	Amplify N-terminal portion of SCZ and combine with C-terminus of *HSFB1*. Use SCZg-F1 as forward primer and pICH41308-SCZg as template.	Assemble two fragments into pICH41308
HSFB1_C-F	TGTGAAGACAATGGAAGGATTGAAATTGTTTGGG	Amplify C-terminus of *HSFB1* and combine with N-terminus of SCZ. Use HSFB1-R as forward primer and pICH41308-HSFB1 as template.	
SCZgNoSTOP-R1	TGTGAAGACAACGAACGTGTAGAAGCAGTCATGAGATTC	Amplify genomic *SCZ* without STOP codon. Use SCZg-F1 as forward primer and pICH41308-SCZg as template.	Assemble into pAGM1287
SCZguDBD-R1	TGTGAAGACAACAGAGAAGTTGTTATGTTTAAAGTAATTAGGA	Generate SCZmDBD. Two PCR reactions: use SCZguDBD-R1 primer with SCZg-F1, SCZguDBD-F4 with SCZg-R4 and pICH41308-SCZg as template.	Assemble into pICH41308
SCZguDBD-F4	TGTGAAGACAATCTGGTGGAGGTGGTGGTGGAGGTACTTATGTATGTAGAATAAAGCTCTCTCCTTT		
SCZguOD-R1	TGTGAAGACAAATCTCTCGTTATCTTCACTCAATGC	Generate SCZmOD. Two PCR reactions: use SCZguOD-R1 primer with SCZg-F1, SCZguOD-F4 with SCZg-R4 and pICH41308-SCZg as template.	Assemble into pICH41308
SCZguOD-F4	TGTGAAGACAAAGAGGAAGGAGGTCTAATACTGTTGGAATGTCGGAACTAGCTCACATGAA		
SCZguRD-R1	TGTGAAGACAACCTGTCTTTCTCATATTACTCTGATCATGA	Generate SCZmRD. Two PCR reactions: use SCZguRD-R1 primer with SCZg-F1, SCZguRD-F4 with SCZg-R4 and pICH41308-SCZg as template.	Assemble into pICH41308
SCZguRD-F4	TGTGAAGACAACAGGGGGTGGTGGTGGTTCGTTGCCTTCTTCTAAGAAGA		
SCZguNES/EAR-R1	TGTGAAGACAAAAGCTTATCTAGTAGATGCTGTCATTCCGTTACCAGCTCCATCAGACTGATCCAACACAAGTCT	Generate SCZmNES/EAR and SCZmRDmNES/EAR. Use SCZg-F1 as forward primer. Use pICH41308-SCZg as template to generate SCZmNES/EAR. Or, use pICH41308-SCZgmRD as template to generate SCZmRDmNES/EAR.	Assemble into pICH41308
SCZguNLS-R1	TGTGAAGACAACCCATATTACTCTGATCATGATGATTAGTG	Generate SCZmNLS. Two PCR reactions: use SCZguNLS-R1 primer with SCZg-F1, SCZguNLS-F4 with SCZg-R4 and pICH41308-SCZg as template.	Assemble into pICH41308
SCZguNLS-F4	TGTGAAGACAATGGGAGGAGGACGTCTCTTCGGTGTTTCTCTTCCATCATCTGGTGGAGGATCACATCATTTCTCAGACCAA		

Supplemental Table S2 continued

HSFB1_swap1_F1	TGTGAAGACAAAATGACGGCTGTGACGGCGGCGCAAAGATCAGTTCCGGCTCCGTTTTTGAC	Amplify from pICH41308-SCZc	Assemble 4 fragments into pICH41308 to generate HSFB1$_{dsOD\&DBD}$
HSFB1_swap1_R1	TGTGAAGACAATTTGTTTTACGGCGGTGGAT		
HSFB1_swap1_F2	TGTGAAGACAACAAAATCGGTGATTGCTTCAAC	Amplify from pICH41308-HSFB1	
HSFB1_swap1_R2	TGTGAAGACAAATCGAACCAGGATTCTTCGAC		
HSFB1_swap1_F3	TGTGAAGACAACGATTGACACGGCGGCG	Amplify from pICH41308-SCZc	
HSFB1_swap1_R3	TGTGAAGACAATGACATGGTTTTGAACAAAGTAGATAATG		
HSFB1_swap1_F4	TGTGAAGACAAGTCAAAGTAAGACCGGAACAAATCG	Amplify from pICH41308-HSFB1	
HSFB1_swap1_R4	TGTGAAGACAAAAGCTTAGTTGCAGACTTTGCTGCTT		
HSFB1_swap2_F1	TGTGAAGACAAAATGACGGCTGTGACGGCG	Amplify from pICH41308-HSFB1	Assemble 2 fragments into pICH41308 to generate HSFB1$_{N}$:SCZ$_{C}$
HSFB1_swap2_R1	TGTGAAGACAACTACCTACCCCCTCCTCTGCT		
HSFB1_swap2_F2	TGTGAAGACAAGTAGAAAGACAAAGCTTTTTGGAGTTT	Amplify from pICH41308-SCZc	
HSFB1_swap2_R2	TGTGAAGACAAAAGCTTAACGTGTAGAAGCAGTCATGAGAT		
HSFB1_swap1_F1	See above	Amplify from pICH41308-SCZc	Assemble 2 fragments into pICH41308 to generate HSFB1$_{dsDBD}$
HSFB1_swap1_R1	See above		
HSFB1_swap1_F2	See above	Amplify from pICH41308-HSFB1	
HSFB1_swap1_R4	See above		
HSFB1_swap2_F1	See above	Amplify from pICH41308-HSFB1	Assemble 3 fragments into pICH41308 to generate HSFB1$_{dsOD}$
HSFB1_swap1_R2	See above		
HSFB1_swap1_F3	See above	Amplify from pICH41308-SCZc	
HSFB1_swap1_R3	See above		
HSFB1_swap1_F4	See above	Amplify from pICH41308-HSFB1	
HSFB1_swap1_R4	See above		
pRPS5AF-BpiGGAG	TGTGAAGACAAGGAGCTCAACTTTTGATTCGCTATTTG	Amplify RPS5A promoter. Use Col-0 genomic DNA as template.	Assembly into pICH41233
pRPS5AR-BpiTACT	TGTGAAGACAAAGTAGGCTGTGGTGAGAGAAACAGA		
aCas9F-BpiAATG	TGTGAAGACAAAATGGATAAGAAGTACTCTATCGGACTC	Recloning aCas9. Plasmid from (Fauser et al., 2014) as template	Assembly into pICH4308
aCas9R-BpiGCTT	TGTGAAGACAAAAGCTCAAACCTTCCTCTTCTTCTTAGG		
SCZ-CRISPRF	CATGAGGACAGATACACACACG	Genotyping *scz*^{CR}	
SCZ-CRISPRR	CTCAAAAACCCCAAATCTCAAG		

Supplemental Table S3. Frequency of phenotypic rescue of *scz-2* and *scz*^{CR} complemented with mScarlet-tagged SCZ variants. Considering that the analyses were performed in segregating T2 lines, presence of a single endodermis and absence of subepidermal root hairs in ~75% of the roots were scored 'fully complementing lines'. Two 'fully complementing lines' were selected for each construct and used for detailed imaging (see Figure 2).

Genotype	Complementing lines		Total lines
	Full	Non	
scz-2;proSCZ::mScarlet-SCZ$_{WT}$	6	9	15
scz^{CR}*;proSCZ::mScarlet-SCZ*$_{WT}$	4	9	13
scz-2;proSCZ::mScarlet-SCZ$_{mDBD}$	0	18	18
scz-2;proSCZ::mScarlet-SCZ$_{mOD}$	0	51	51
scz-2;proSCZ::mScarlet-SCZ$_{mRD}$	3	9	12
scz-2;proSCZ::mScarlet-SCZ$_{mNES/EAR}$	5	8	13
scz-2;proSCZ::mScarlet-SCZ$_{mRDmNES/EAR}$	0	6	6
scz^{CR}*;proSCZ::mScarlet-SCZ*$_{mRDmNES/EAR}$	0	17	17
scz-2;proSCZ::mScarlet-SCZ$_{mNLS}$	4	12	16
scz-2;proSCZ::mScarlet-SCZ$_{+SRDX}$	3	5	8
scz-2;proSCZ::mScarlet-SCZ$_{N}$*:HSFB1*$_{C}$	3	13	16
